# Combination of RGD Compound and Low-Dose Paclitaxel Induces Apoptosis in Human Glioblastoma Cells

**DOI:** 10.1371/journal.pone.0037935

**Published:** 2012-05-24

**Authors:** Ming-Wei Chang, Jem-Mau Lo, Hsueh-Fen Juan, Hsin-Yi Chang, Chun-Yu Chuang

**Affiliations:** 1 Department of Biomedical Engineering and Environmental Sciences, National Tsing Hua University, Hsinchu, Taiwan; 2 Institute of Molecular and Cellular Biology, National Taiwan University, Taipei, Taiwan; 3 Department of Life Science, National Taiwan University, Taipei, Taiwan; National Center for Scientific Research Demokritos, Greece

## Abstract

**Background:**

Integrins are a family of transmembrane adhesion proteins that mediate cell adhesion and intracellular signaling. Integrin-αvβ3 is expressed on the surface of human glioblastoma cells, and can be further induced by chemical stress. The Arg-Gly-Asp (RGD) motif-containing peptides are specifically bound to integrin-αvβ3, and to inhibit neovasculature underlying competition to normal extracellular matrix proteins. This study employed two types of RGD peptides, cyclic RGD (c(RGDyK)) and bi-cyclic RGD (E[c(RGDyK)]_2_) peptide, to human glioblastoma U87MG cells with combination of low dose Paclitaxel (PTX) pre-treatment to augment therapeutic activity for RGD peptide-induced apoptosis.

**Principal Findings:**

Human glioblastoma U87MG cells were treated with RGD peptides in the absence or presence of initial exposure to low-dose 10 nM PTX. Results showed that integrin-αvβ3 expressing on the surface of U87MG cells was induced by 10 nM PTX pre-treatment for 12 hrs. Additionally, the U87MG cells pre-treated with PTX and followed by RGD peptides exhibited greater expression of caspases-3, -8 and -9 than those merely treated with single agent of PTX or RGD peptide. Furthermore, the caspase-3, -8 and -9 inhibitor presented significant protection against E[c(RGDyK)]_2_ peptide induced U87MG programmed cell death. The increased expression of PTX-induced integrin-αvβ3 was correlated with the enhanced apoptosis in U87MG cells.

**Conclusions:**

This study provides a novel concept of targeting integrin-αvβ3 with RGD peptides in combination with low-dose PTX pre-treatment to improve efficiency in human glioblastoma treatment.

## Introduction

Glioblastoma multiforme (GBM) is a common type of human brain tumor composed of poorly differentiated astrocytes [1]. GBM is notorious for its highly invasive behavior and usually responds poorly to conventional cytotoxic therapy. At present, the therapeutic strategies for GBM include radiation therapy or surgery in combination with anti-cancer drugs (e.g., Temozolomide, Gliadel wafer, and Carmustine) [2], [3]. Unfortunately, gliomas are resistant to standard chemotherapeutic and radiation therapeutic approaches because they exhibit infiltrative growth patterns [4]. Therefore, the development of an optimal molecular targeting therapy is an urgent need in GBM therapy.

Programmed cell survival or death directly relies on the interactions between integrin receptors and extracellular matrixs (ECMs). Integrins are a class of cell adhesion molecules that mediate the cell-ECM interaction and regulate cell survival, adhesion, migration, proliferation, and differentiation. The non-covalent assembly of eighteen α and eight β subunits forms 24 integrin heterodimers [5], [6]. These α and β heterodimers modulate cellular signal transduction by forming between the extracellular and cytoplasmic domain to dock with cytoskeleton, microfilament associated proteins and most ECM proteins (e.g., fibronectin, vitronectin, laminin and osteopontin) [5], [7]. As a primary receptor of cell-ECM adhesion molecules, integrin-αvβ3 acts as a crucial transducer in regulating cell death signaling [7], [8]. An abundance of integrin-αvβ3 has been found in human malignant glioblastoma, melanoma, breast tumors, metastatic prostate tumors, and ovarian tumors [1], [9]. However, the integrin-αvβ3 receptor protein is not persistently expressed on various tumor cells, and is often deficient in normal cells [10]. For example, human melanoma A375 cells express significantly higher integrin-αvβ3 than human breast cancer MDA-MB 435 cells and human prostate tumor DU145 cells [11]. The progression of tumor invasiveness and metastasis is positively correlated with the expression levels of integrin-αvβ3 in human prostate cancer LNCaP cells [12]. When antagonized by RGD peptides, the integrin-αvβ3 receptor protein has the capability to program cell death through cell apoptosis or neovasculacture inhibition in human glioblastoma (10 mM RGD peptides), lung fibroblasts (0.8 mM) and breast carcinoma MCF-7 cells (1 mM) [1], [13], [14], [15]. The RGD pentapeptide Cilengitide (EMD 121974, Merck), an antagonist of integrin-αvβ3, is currently used to treat tumors by inhibiting angiogenesis in clinical phase 3 testing for GBM, and phase 1, 2 trials for metastatic squamous cell carcinoma and advanced non-small cell lung cancer [16], [17], [18]. Unfortunately, the single agent of Cilengitide only has antitumor benefit and toxicity in newly diagnosed GBM patients, but is well tolerated in recurrent GBM patients.

Paclitaxel (PTX) is the broadest-spectrum anticancer agent isolated from the Pacific Yew tree Taxus brevifolia, and is currently used in the treatment of head, neck, lung, breast, ovarian and bladder cancers [14], [19]. PTX is an anticancer agent due to its efficient induction of apoptosis. PTX interferes with microtubule assembly by binding and stabilizing β-tubulin in the G2/M phase of the cell cycle [14]. However, the clinical application of PTX are usually limited by the serious side effects of drug cytotoxicity (bruising or bleeding, anemia and peripheral neurotoxicity) and its low aqueous solubility [14]. Therefore, this study used a low-dose of PTX (10 nM) as a trigger to activate increased expression of integrin-αvβ3 in human glioblastoma multiforme U87MG cells. In the case of multiple myeloma, the malignant cells can express β1 integrins and utilize them to adhere to bone marrow stromal cells promoting survival and drug resistance [20]. Lorger et al. [12] demonstrated that the activated tumor cell integrin-αvβ3 supports efficient brain metastatic growth through continuous up-regulation of vascular endothelial growth factor protein under normoxic in CB17/SCID mice. The increased integrin-αvβ3 expression is involved in and mediates the invasion/metastasis of tumor cells [21]. In general, normally-expressed integrin-αvβ3 on cells is involved in cell-cell interactions and signal transduction. Integrin-αvβ3 is highly expressed on activated endothelial cells and tumor cells but not on quiescent cells. In the case of the later, this presents a dichotomy, i.e., the increased expression allows for enhanced invasiveness and metastatic potentials yet also makes the cell more likely targets for an agent against the integrin during tumor imaging or anti-cancer therapy [22]. In this study, the over-expression of integrin-αvβ3 supplies more epitope binding sites for improving RGD peptides binding efficiency and intervening downstream cellular signaling.

Programmed cell death through caspase activation includes two major pathways - the release of cytochrone c from mitochondria (intrinsic pathway) or the ligand binding with death receptors (extrinsic pathway) [23], [24]. However, the underlying mechanism of RGD peptide-integrin-αvβ3 mediated brain tumor cell death remains unclear. Therefore, this study investigates the apoptotic potential and the gene network of the caspase cascade in U87MG cells undergoing RGD peptides specific-targeting to integrin-αvβ3 that is enhanced by low-dose PTX treatment.

## Materials and Methods

### Cell culture

Human glioblastoma multiforme U87MG cells (ATCC HTB-14) were cultured in minimum essential medium with 1 nM sodium pyruvate (Invitrogen, Carlsbad, CA) and 10% fetal bovine serum (BSR Bio, Walkerville, Australia) in a cell incubator with 5% CO_2_ at 37°C.

### Docking of RGD peptides

**Figure 1:**
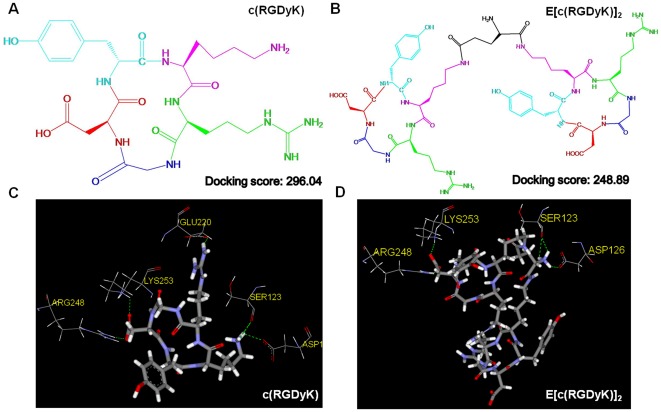
Schematic illustration of chemical structures and docking stimulation outlining. (A, C) c(RGDyK) and (B,D) E[c(RGDyK)]_2_ peptides, respectively.

In this study, c(RGDyK) and E[c(RGDyK)]_2_ peptides were individually used as an antagonist to integrin-αvβ3. Both c(RGDyK) and E[c(RGDyK)]_2_ peptides were kindly supplied by Dr. J. M. Lo (National Tsing Hua University, Taiwan). The chemical structure and the docking score of RGD peptides were shown in Fig. 1. A receptor molecular model was simulated by docking experiment to investigate the avidity of RGD peptides to integrin-αvβ3. A shape-based docking algorithm was used to fit the RGD peptides within the integrin-αvβ3 receptors and conformation flexibility, and the score was provided depending on Ligscoring function [25]. The docking protocol was described as previous studies [25], [26].

### Cell treatment

The c(RGDyK) and E[c(RGDyK)]_2_ peptides were prepared by DMSO in 1 mM stock. The U87MG cells (2×10^5^) were seeded into 6-cm culture plate and then treated with 10 nM PTX for 12 hrs or 100 µM of c(RGDyK)/E[c(RGDyK)]_2_ for 24 hrs. For some experiments, the cells were pre-treated with 10 nM PTX for 12 hrs, then washed three time with PBS and treated with 100 µM c(RGDyK)/E[c(RGDyK)]_2_ for a further 24 hrs. The final Cremophor EL and DMSO (vehicle respectively for PTX and RGD peptides) concentration in the treated culture was <0.1% in the experiment and there was no vehicle effect on viability (data not shown). The preparation of treating reagent as described in the next paragraph.

### Determination of integrin-αvβ3 expression

This study sought to determine if integrin-αvβ3 expression of U87MG cells was altered in response to the 12 hr pre-treatment with 10 nM PTX. Monoclonal anti-human integrin-αvβ3 (CD51/61)-phycoerythrin antibody and anti-human IgG (Fc specific) were purchased from R & D systems Inc. (Minneapolis, MN) and Sigma (St. Louis, MO), respectively. PTX (ScinoPharm, Tainan, Taiwan) was diluted with Cremophore EL (Sigma) for stock concentration 1 mM. Following the above-outline treatments, the U87MG cells were washed with cold PBS for three times. The cells (1×10^5^) were then incubated with 1 µg anti-human IgG for 15 min at room temperature to block non-specific binding, then incubated in 10 µl monoclonal anti-human integrin-αvβ3 (CD51/61)-phycoerythrin antibody at 4°C for 45 min, and determined by flow cytometry (Partect, Münster, Germany) using WinMDI 2.8 software. The U87MG cells incubated in 1 µg of anti-human IgG without anti-human integrin-αvβ3 staining as the isotype control.

### Immunofluorescence staining of integrin-αvβ3

U87MG cells were fixed in 4% paraformaldehyde for 10 min at ambient temperature, and incubated in 3% bovine serum albumin (PBST) for 30 min to block non-specific antibody binding. The fixed U87MG cells were stained using monoclonal anti-integrin-αvβ3 Ab (1∶500) (Abcam, Cambridge, UK) at 4°C overnight and 1 hr incubation of anti-mouse Texas red Ab (1∶500) (Abcam) in dark. DAPI (1∶1000) (Sigma) was used to localize nuclei of U87MG cells. An Axiovert 40 CFL microscope (Carl Zeiss, St. Louis, MO) was used to determine the signals of Texas red and DAPI for integrin-αvβ3 and nuclei staining.

### Viability assay

The cell viability was assessed by 3-(4,5-dimethylthiazol-2-yl)-2,5-diphenyltetrazolium bromide (MTT; Sigma). The U87MG cells were seeded at density of 5×10^3^ cells per well in a 96-well plate and treated with a series concentration of RGD peptides (50–200 µM). Cells were incubated in MTT (0.5 mg/ml) containing culture medium at 37°C for 4 hrs. To each well was added 100 µl DMSO and mixed completely to dissolve MTT. Absorbance was measured at 570 nm with background subtraction at 650 nm by Versamax microplate reader (Molecular devices Inc., Toronto, Canada).

### Determination of cell cycle and apoptosis

The progression of cell cycle and apoptosis was determined by propedium iodine (PI; Sigma) and Annexin V-FITC (Invitrogen). The U87MG cells were harvested by trypsinization method, washed with PBS once, and fixed by 70% cold ethanol, and storage at −20°C for 1 hr, followed by incubation with PI/Triton X-100/RNase A staining solution (0.1% Triton X, 0.2 mg/ml RNase A and 20 µg/ml PI in PBS) at room temperature for 30 min. The staining cells (10,000 cells) of each sample were detected by flow cytometry (Partect), and the percentage of cell cycle phases and apoptosis was analyzed using FlowMax software.

### Reverse transcription polymerase chain reaction (RT-PCR)

Total RNA was isolated from U87MG cells using RNA Trizol (Invitrogen), and cDNA was synthesized from total RNA by a high-capacity cDNA reverse transcription kit (Applied Biosystems Inc.). In brief, 3 µg RNA was added 1.0 µl MultiScribe™ reverse transcriptase (50 unit/µl), 2.0 µl 10× RT random primers, 0.8 µl 20× dNTP mix, 2.0 µl 10× RT buffer and RNase free water in a 0.2 ml PCR tube, and subsequently amplified by PCR with one cycle of 20°C 10 min, 37°C 120 min and 85°C 5 sec.

### Quantitative real time PCR

The gene expression of caspases-3, -8, -9, -10 and -12 were determined by quantitative real-time PCR. Each 20 µl reaction contained 6.8 µl RNase free water, 0.4 µl for each forward and reverse primers, 0.4 µl ROX reference dye, 10 µl SYBR premix Ex Taq (TaKaRa Bio Inc., Shiga, Japan), and 2 µl (100 ng) template cDNA. Primers used in this study were shown in Table 1. The cycling condition of ABI Prism 7300 real-time PCR system (Applied Biosystems Inc.) were 50°C for 2 min, 95°C for 10 min, and 95°C 15 sec, 55°C 30 sec, 72°C 45 sec for 40 amplification cycles, with extension 95°C 15 sec and 60°C 1 min. The relative level of mRNA expression was analyzed using comparative method by SDS 1.4 software normalized to the endogenous housekeeping gene β-actin.

**Table 1:** Primers used in real-time PCR analysis.

Gene	Sense primer (5′→3′)	Anti-sense primer (5′→3′)
Caspase 3	TGGTTCATCCAGTCGCTTTGT	AATTCTGTTGCCACCTTTCGG
Caspase 8	AAAAGCAAACCTCGGGATAC	CCAAGTGTGTTCCATTCCTGTC
Caspase 9	TGGACATTGGTTCTGGAGGATT	CACGGCAGAAGTTCACATTGTT
Caspase 10	GGCTATGTATCCTTTCGGCA	CCCTGTTTGTCCACTCTTCG
Caspase 12	GCCTCAACATCCGCAACAA	ATCTCACATCCCCAAAAGGTC

### Neutralized effect of caspase inhibitors on RGD-induced apoptosis in U87MG cells pre-treated with low-dose PTX

The inhibitors of caspase-3 (Z-DEVD-FMK) (Biovision, San Francisco, CA), caspase-8 (Z-IETD-FMK) and caspase-9 (Z-LEHD-FMK) (R&D systems, Minneapolis, MN) were used to determine which caspases were involved in RGD peptide-induced apoptosis with low dose PTX pre-treatment. Caspase inhibitors were dissolved in dimethyl sulfoxide (20 mM). U87MG cells were seeded at 2×10^5^ cells/ml and pre-incubated with 10 µM caspase inhibitor for 3 hrs followed by 12 hr 10 nM PTX pre-treatment and 24 hr E[c(RGDyK)]_2_ peptide. The same amount of DMSO as treatment groups was used in the control group. Three independent experiments were performed for determination of caspase gene expression.

### Statistical analysis

Results were obtained through at least three separate experiments. Data are reported as means ± SD. All statistical analysis was conducted by Microsoft Excel Statistic Suit. Statistically significant differences among groups were determined using Student's t test (p<0.05).

## Results

### RGD peptide docking simulation

This study assessed the interaction of RGD peptides with integrin-αvβ3 through the docking simulation performing on the Discovery Studio 1.6 environment. Results showed that the scores of docking simulation were 296.04 and 245.89 for c(RGDyK) and E[c(RGDyK)]_2_ peptides, respectively (Fig. 1). The output convergent conformations demonstrated that the RGD peptides used in this study had reliable binding affinity towards the integrin interface.

### PTX induced expression of integrin-αvβ3

**Figure 2:**
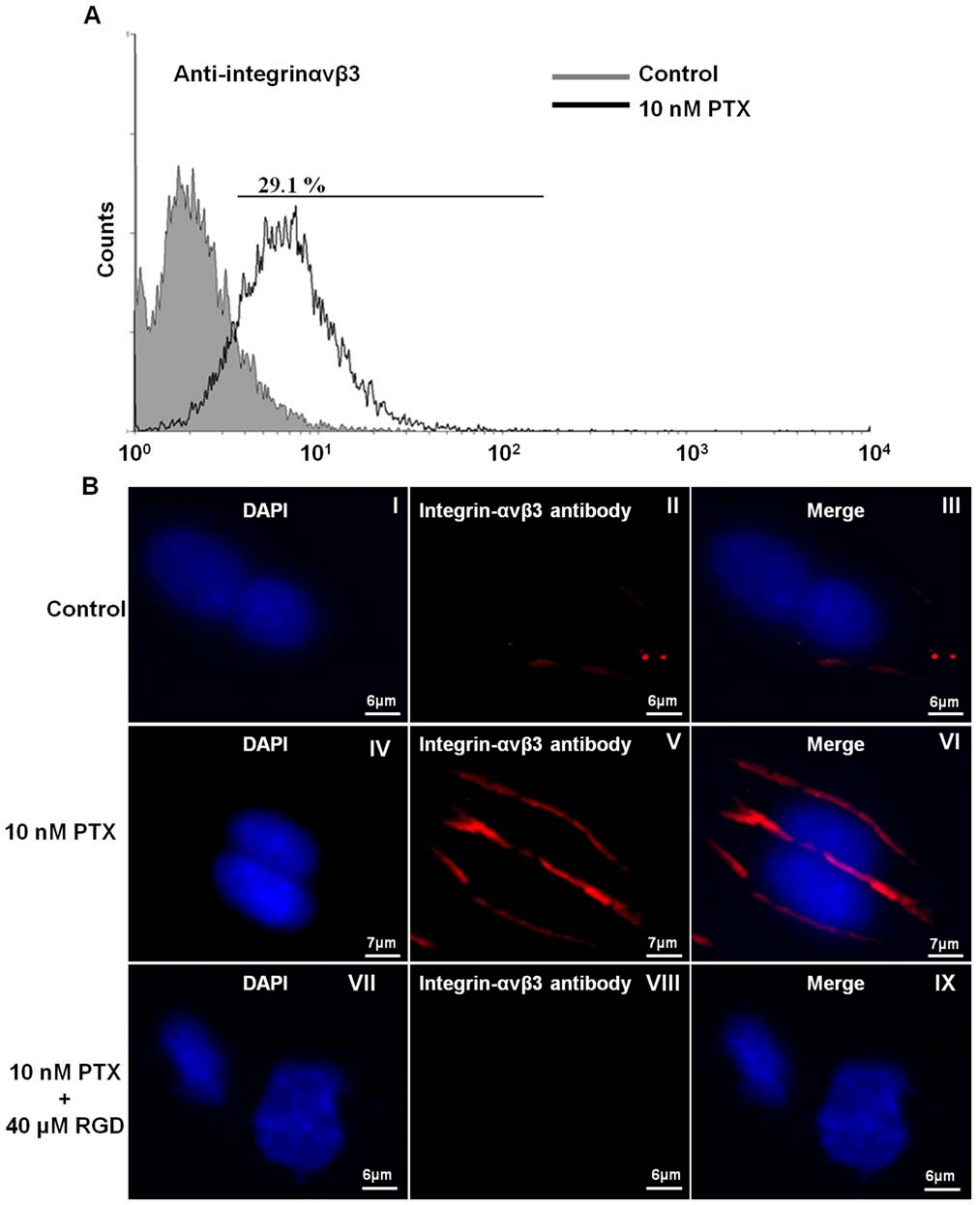
Expression of surface integrin-αvβ3 in U87MG cells with the treatment of 10 nM PTX. (A) Cytometric analysis of the expression of integrin-αvβ3 was determined by specific integrin-αvβ3 antibody staining. The expression of integrin-αvβ3 in U87MG cells treated with 10 nM PTX for 12 hrs was higher than control group. (B) Immunofluorescent detection of integrin-αvβ3 and nuclei was respectively stained by anti-integrin-αvβ3 antibody (Texas red) and DAPI (blue). Integrin-αvβ3 was obviously localized in the cell periphery. (I-III) indicated the U87MG cell isotypic control group. (IV-VI) Integrin-αvβ3 of U87MG cells was induced after 12 hr 10 nM PTX. (VII–IX) U87MG cells were pre-incubated with E[c(RGDyK)]_2_ peptide to antagonized PTX-induced integrin-αvβ3 expression.

The expression of integrin-αvβ3 on U87MG cell surface was determined using flow cytometry with anti- integrin-αvβ3 antibody. U87MG cells exposed to 10 nM PTX for 12 hr had a significant enhanced expression of integrin-αvβ3 (Fig. 2A). To examine whether low-dose 10 nM PTX treatment induced integrin-αvβ3 over-expression, the immunofluorescent detection of integrin-αvβ3 and DAPI (4′,6-diamidino-2-phenylindole) staining were performed. The results in Fig. 2B(IV-VI) showed that integrin-αvβ3 was induced by 10 nM PTX and localized around the cell membrane. The U87MG cells were incubated E[c(RGDyK)]_2_ peptide at 37°C and then treated with 10 nM PTX to evidence RGD peptide antagonized PTX-induced integrin-αvβ3 epitope. The result showed U87MG cells were entirely blocked by RGD peptide that meant the RGD peptide was specific binding to integrin-αvβ3 binding (Fig. 2B(VII–IX)).

### Cytotoxic and apoptotic effects of PTX and RGD peptides on U87MG cells

**Figure 3:**
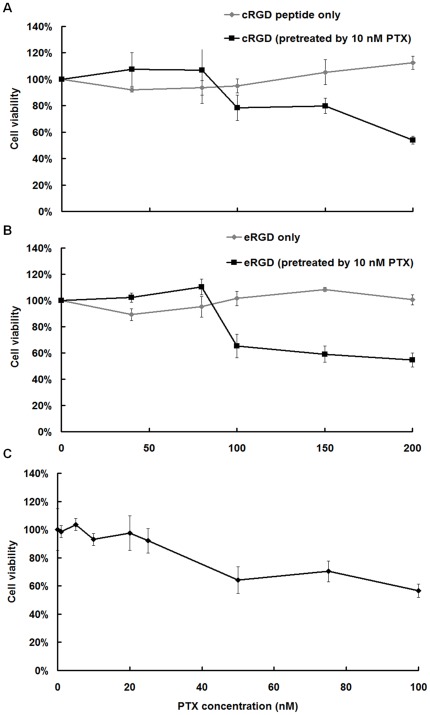
Cytotoxicity of U87MG cells treated with RGD peptide or the combination of PTX and RGD peptide was quantified using MTT assay. (A) Cell viability of c(RGDyK) peptide or combination pre-treated by 10 nM PTX in U87MG cells. (B) Cell viability of E[c(RGDyK)]_2_ peptides or combination pre-treated by 10 nM PTX in U87MG cells. Cell viability was decreased at 100 µM c(RGDyK) or E[c(RGDyK)]_2_ peptide. (C) Cell viability of a serial concentration of PTX for 24 hr in U87MG cells. The results of triplicate experiments are expressed as mean ± standard deviation.

**Figure 4:**
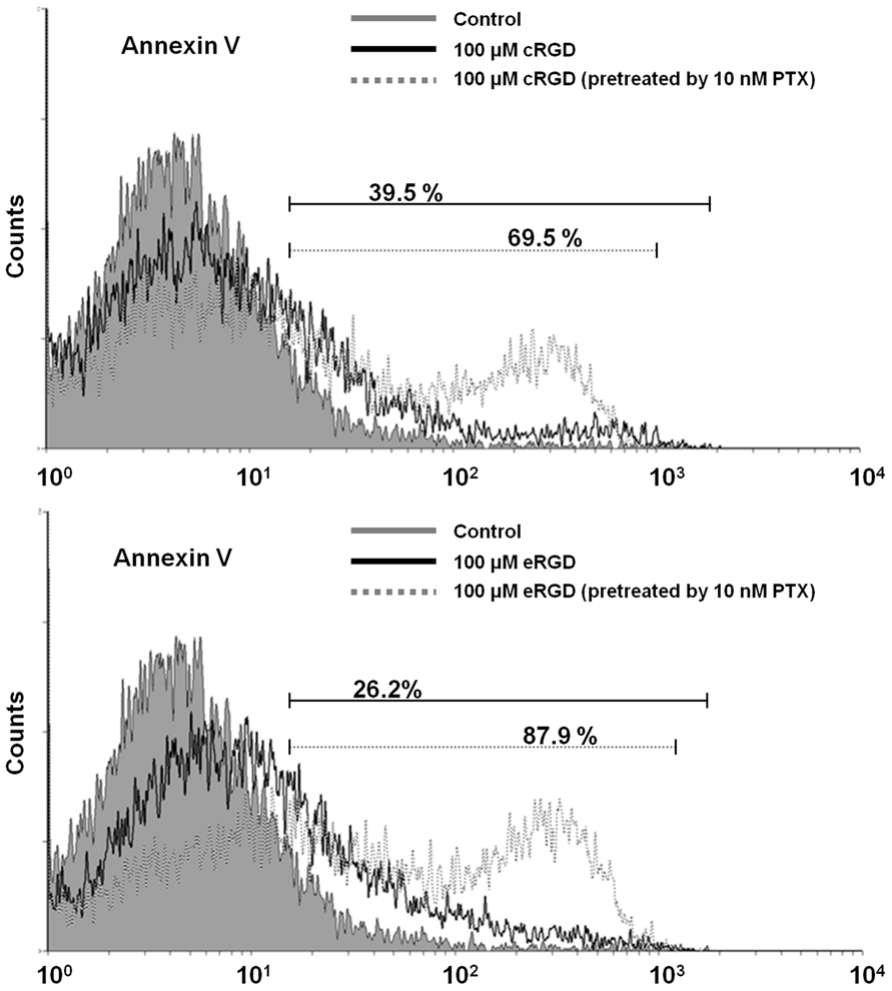
Cytometric analysis of apoptosis events in U87MG cells treated with PTX, c(RGDyK)/E[c(RGDyK)]_2_ peptides or the combination of PTX and c(RGDyK)/E[c(RGDyK)]_2_ peptides. Determination of apoptosis was using Annexin-V-FITC staining and FACS detector. The occurrence of programmed apoptosis was obviously in U87MG cells pre-treated with 10 nM PTX followed by 100 µM (A) c(RGDyK)/(B) E[c(RGDyK)]_2_ peptides. Triplicate experiments were obtained for analysis.

The results of the MTT assay indicated that c(RGDyK)/E[c(RGDyK)]_2_ (0, 40, 80, 100, 150, 200 µM) had no cytotoxicity to U87MG cells (Fig. 3A and 3B). This study used low-dose PTX to induce over-expression of integrin-αvβ3. Cell viability of U87MG cells was decreased after 12 hr 10 nM PTX pre-treatment and following exposure to 100 µM c(RGDyK)/E[c(RGDyK)]_2_ peptides for 24 hrs (Fig. 3A and 3B). According to the results of cell viability using the MTT assay, the predictive LD50 of PTX in this study was higher than 100 nM (Fig. 3C). The applied low-dose of 10 nM PTX for 12 hr had no effect on cell death in U87MG cells (Fig. 3C). The apoptotic percentage of U87MG cells pre-treated with 10 nM PTX for 12 hr and then 100 µM c(RGDyK)/E[c(RGDyK)]_2_ for 24 hr was higher than those with 24 hr c(RGDyK)/E[c(RGDyK)]_2_ treatment alone (69.5 [±0.8%] vs. 39.5 [±0.6%], Fig. 4A; 87.9 [±1.3%] vs. 26.2 [±0.9%], Fig. 4B).

### Gene expression of caspase signaling regulated by PTX and RGD peptide

**Figure 5:**
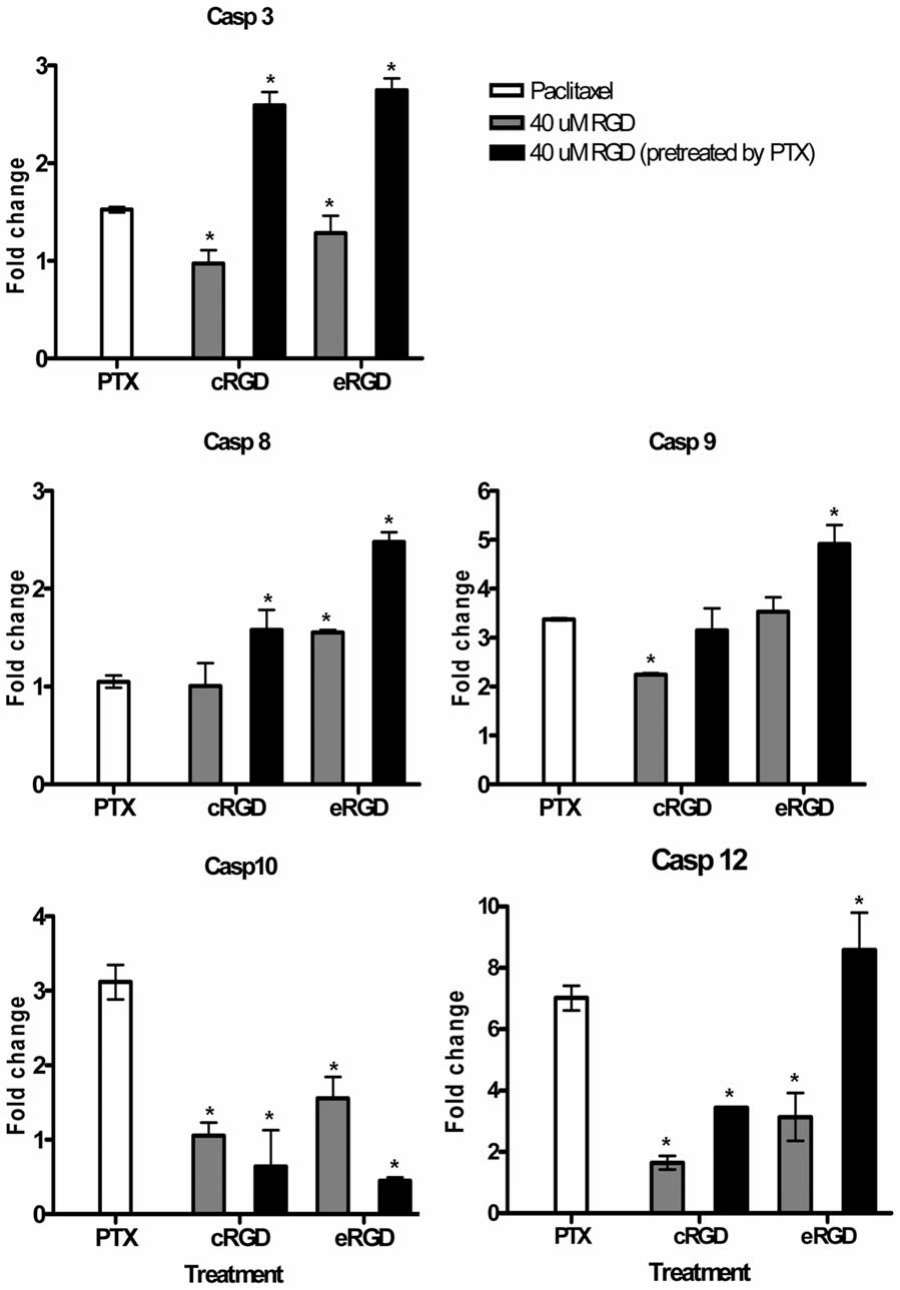
Quantitative expression of caspase genes in U87MG cells treated with PTX, c(RGDyK)/E[c(RGDyK)]_2_ peptides or the 12 hr PTX pre-treatment following with c(RGDyK)/E[c(RGDyK)]_2_ peptides exposure. U87MG cells were treated with 40 µM c(RGDyK)/E[c(RGDyK)]_2_ peptides for 24 hrs with or without 10 nM PTX pre-treatment for 12 hrs. The mRNA expression of (A) caspase-3, (B) caspase-8, (C) caspase-9, (D) caspase-10, and (E) caspase-12 was determined using real-time PCR. The increased levels of caspases -3, -8 and -9 were apparent in the pre-treatment with PTX for 12 hrs and 24 hr 40 µM c(RGDyK)/E[c(RGDyK)]_2_ peptides. (fold changes of caspases -3, -8 and -9; PTX+c(RGDyK) vs. PTX alone: caspase-3 = 2.4 [±0.1] vs. 1.5 [±0.02], caspase-8 = 1.5 [±0.2] vs. 1.0 [±0.1], and caspase-9 = 3.1 [±0.3] vs. 3.4 [±0.02]; PTX+E[c(RGDyK)]_2_ vs. PTX alone: caspase-3 = 2.7 [±0.1] vs. 1.5 [±0.02], caspase-8 = 2.4 [±0.1] vs. 1.0 [±0.1], and caspase-9 = 5.0 [±0.4] vs. 3.4 [±0.02]). The bars are represented the mean fold change of at least three independent experiments. Asterisk indicates significant at p<0.05 compared with 10 nM PTX alone.

**Figure 6:**
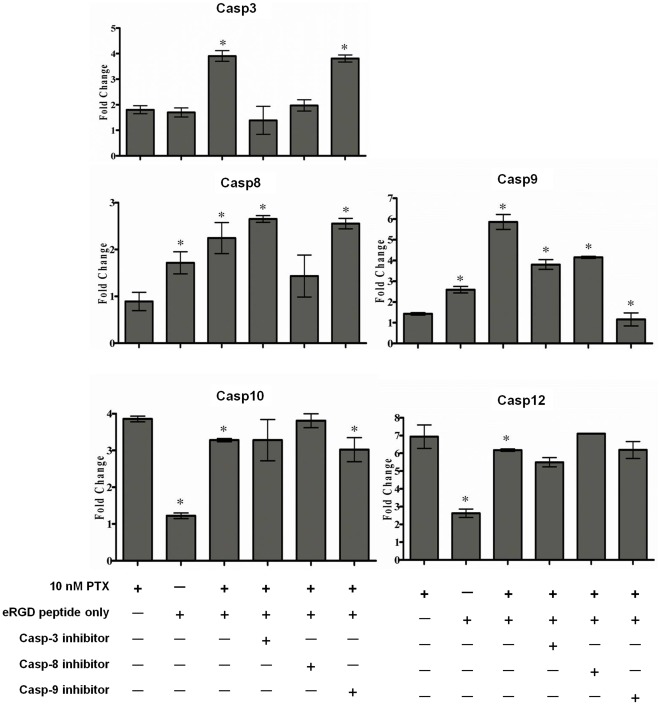
Neutralized effect of caspase-3, -8 and -9 inhibitors on the caspase mRNA expression induced by PTX pre-treatment and E[c(RGDyK)]_2_ peptide. U87MG cells were respectively pre-incubated with inhibitors of caspase-3 (Z-DEVD-FMK), caspase-8 (Z-IETD-FMK) or caspase-9 (Z-LEHD-FMK) and followed by 10 nM PTX pre-treatment for 12 hr and 40 µM E[c(RGDyK)]_2_ treatment for 24 hr. The mRNA expression of (A) caspase-3, (B) caspase-8, (C) caspase-9, (D) caspase-10, and (E) caspase-12 was determined using real-time PCR. Eventually, either caspase-3, -8 or -9 inhibitor effectively to suppress the expression of caspase-3, -8 and -9. Asterisk indicates significant at p<0.05 compared with 10 nM PTX alone.

This study applies low-dose PTX and RGD peptide to mediate the programmed brain tumor cell death, and further investigated the reconstruction of caspase gene network. The results of real-time PCR showed that the dose of 10 nM PTX induced the augment expression of caspase-3, -8, -9, -10 and -12 genes (Fig. 5). Exposure of the U87MG cells to to 40 µM c(RGDyK) peptide following a 10 nM PTX pre-treatment increased the expression of caspase-3, -8 and -9 compared to that seen following treatment with 10 nM PTX alone (fold-changes respectively of: caspase-3 = 2.4 [±0.1] vs. 1.5 [±0.02], respectively; caspase-8 = 1.5 [±0.2] vs. 1.0 [±0.1]; and caspase-9 = 3.1 [±0.3] vs. 3.4 [±0.02]). The mRNA expression of caspase-3, -8, and -9 achieved 2.7 [±0.1] vs. 1.5 [±0.02], 2.4 [±0.1] vs. 1.0 [±0.1], and 5.0 [±0.4] vs. 3.4 [±0.02] -fold changes in response to the exposure of 40 µM E[c(RGDyK)]_2_ peptide with 10 nM PTX pre-treatment that compared to 10 nM of PTX single agent treatment, respectively. Definitely, the E[c(RGDyK)]_2_ peptide was more efficient to cause apoptosis for U87MG cells underlying its double side wing structure. Furthermore, to determine whether the changed expression of caspase genes was underwent programmed apoptosis, the specific inhibitors of caspase-3, -8 and -9 were used to elucidate this extrinsic caspase pathway. The expression of caspase-3, -8, and -9 genes was apparently suppressed in U87MG cells pre-incubated with, respectively, Z-DEVD-FMK, Z-IETD-FMK and Z-LEHD-FMK, and then underwent the 10 nM PTX and 40 µM E[c(RGDyK)]_2_ peptide treatment regimen (Fig. 6; fold change w/o vs. with inhibitor: 3.9 [±0.2] vs. 1.4 [±0.5] for caspase-3, 2.4 [±0.3] vs. 1.4 [±0.4] for caspase-8, 5.9 [±0.4] vs. 1.1 [±0.3] for caspase-9).

## Discussion

This study used low-dose PTX to activate inegrin-αvβ3 expression and antagonized to RGD peptides for programmed cell death. The two RGD peptides, c(RGDyK) and E[c(RGDyK)]_2_, used in this study have the affinity for integrin-αvβ3. The docking simulation showed that c(RGDyK) peptide had higher avidity to integrin-αvβ3 than E[c(RGDyK)]_2_ peptide. This might be due to the stereo structure of E[c(RGDyK)]_2_ peptide, which consists of two side wings to neutralize the total docking score. The two side wings of E[c(RGDyK)]_2_ peptide provided better flexibility to attach integrin-αvβ3 expressing in U87MG cells. The results of MTT assay showed that E[c(RGDyK)]_2_ peptide had greater cytotoxicity than c(RGDyK) peptide. Additionally, as expected, E[c(RGDyK)]_2_ was more efficient in activating caspase signaling for programmed cell death than c(RGDyK) peptide.

Tumoricidal agents such as methylating procarbazine, chloroethylating BCNU (1,3-bis(2-chloroethyl)-1-nitrosourea; carmustine), and CCNU (1-(2-chloroethyl)-3-chlohexyl-1-nitrosourea; lomustine) have been used in the clinical treatment of human brain tumors [27]. Nonetheless, the outcome remains restricted due to the DNA repair activities and the drug efflux system as inferred from the presence of P-glycoprotein in brain capillaries [28]. Thus, this study used PTX as an anticancer agent to avoid possible interference with inertia genotype. PTX-induced programmed cell death is directed by death receptor ligand binding (e.g. Fas-associated death domain protein; FADD), cell cycle interventions, and characteristic p53-independent [14], [29]. In the past two decades, there have been various medical advancements in drug innovation and treatment methods. Several novel agents targeting DNA base pair for disrupting tumorgenesis of glioblastoma have been developed. Previous studies reported that RGD peptides can specifically bind to integrin-αvβ3 in tumor cells but not in normal tissue cells [30], [31]. However, therapeutic targeting strategies are usually restricted by the amount of substance (receptor) for ligand (peptide drug) attachment and the influence of non-targeting cells to confine cytotoxicity efficiency toward tumor cells.

This study examined the role of integrin-αvβ3 in response to low-dose (10 nM) PTX using the brain tumor U87MG cell line model. The cell surface expression of integrin-αvβ3 increased dramatically in U87MG cells pre-treated with 10 nM PTX for 12 hr compared to those in the control group. This integrin clustering effect was due to the reorganization of actin filament into large stress fibers as the inside-out signaling [7]. PTX is often used against various tumors in clinical applications. However, the dosimetry studies of PTX used to treat human brain cancer derived cell lines with a wide tolerant range which depends on the duration and concentration of PTX exposure [32]. The LD90 of PTX is defined 250–300 nM for 1 hr treatment in human glioblastoma brain tumor [14], [33]. In this study, low-dose (10 nM) PTX was not used directly to program cell death but acted as a stressor to increase the expression of integrin-αvβ3 on cell membranes. As shown in Fig. 2A and 2B (IV), integrin-αvβ3 of U87MG cells were likely to be induced after 12 hrs 10 nM PTX treatment. Moreover, the results of immunostaining in this study showed U87MG cells were entirely blocked by E[c(RGDyK)]_2_ peptide which is used in this study was specific binding to integrin-αvβ3 binding (Fig. 2B(IX)). From the basis studies using electron microscopy, NMR, FRET (Förster resonance energy transfer) and crystallography, integrins appearing the bent conformation and low affinity state are determined as the inactive form [34], [35]. The increased integrin-αvβ3 complexes can form catch bonds (become strengthened) under further stimulation that is induced from the extracellular or intracellular end of molecules [36], [37]. This natural rising phenomenon suggested the opportunity to solve the deficiency of integrin-αvβ3 in various tumor cells thereby improving therapeutic effect. The results of flow cytometry showed that U87MG cells treated with 10 nM PTX (29.93%) had cell cycle arrest in G2/M phase in comparison with the control group (19.45%) (data not shown). This confirmed the mitotic inhibition characteristics of low-dose PTX (10–100 nM) in an arrest at G2/M phase of the cell cycle [6].

Previous studies explored the mechanisms through which integrin mediates cell death showing that the induced apoptosis is correlated to the subcellular localization of activated caspase cascade [1], [5], [33]. Based on their homology in amino acid sequence, the initiator caspase signaling molecules caspase-8 and -9, are responsible for activation of caspase cascade and to induce the effector caspase-3 for the actual execution of apoptosis (so-called extrinsic pathway) [38]. In this study, in the presence of 40 µM RGD peptide in U87MG cells, the mRNA levels of caspase-3, -8 and -9 exhibited up to 3.5-fold increase. These caspase cascades were involved in death receptor-mediated pathways. Previous studies showed that blocking integrin-αvβ3 via RGD peptides induces cell death in human endothelial cells through activation of caspase-3, -8 and -9, FADD (Fas-Associated protein with Death Domain), tumor necrosis factor-α and TRAIL (TNF related apoptosis inducing ligand) pathways and is correlated to the down-regulation of FAK (focal adhesion kinase) expression in tumor cells [39], [40]. In addition, the RGD sequence contains the large subunit of caspase-3 and the small subunit of caspase-8, beginning at positions R144 and R435, respectively [41]. The equivalent dosage of PTX in clinical applications required to induce apoptosis depends on the release of cytochrome c from mitochondrial (intrinsic pathway) or the activation of caspase-10 and -12, and their downstream signaling pathways caspase-6, -8 and -3 [14], [19], [42], [43]. In addition, the low-dose PTX induced cell cycle arrest and suppressed the dynamics of microtubule in U87MG cells, which facilitated the activation of caspase-10 and -12, and promoted the following caspase cascade. Caspase-10 and -12 played an essential role of initiator in apoptosis by PTX pre-treatment. The viability assay in this study suggested that there was no cytotoxicity effect up to 200 µM of RGD peptide in a single treatment, but the decreased cell viability appeared at 100 µM RGD peptide exposure after 10 nM PTX pre-treatment. These results suggested that the RGD peptide alone was not sufficient enough to induce cell apoptotic responses immediately, but the combination of 40 µM RGD peptide with 10 nM PTX led to the up-regulation of caspase-3, -8, and -9 for the apoptotic process. In a brief summary, U87MG cells pre-treated wiht 10 nM PTX following with treatment of 40 µM RGD peptide potentially boost for programmed cell apoptosis than 10 nM PTX or 40 µM RGD peptide treatment alone. Furthermore, for U87MG cells exposed to E[c(RGDyK)]_2_ with 10 nM PTX pre-treatment showed the efficiency for up-regulation caspase genes -3, -8 and -9 than cells exposed to c(RGDyK) combined 10 nM pre-treatment (E[c(RGDyK)]_2_+PTX vs. c(RGDyK)+PTX caspase-3 = 2.7 [±0.1] vs. 2.4 [±0.1], respectively; caspase-8 = 2.4 [±0.1] vs. 1.5 [±0.2]; and caspase-9 = 5.0 [±0.4] vs. 3.1 [±0.3]).

**Figure 7:**
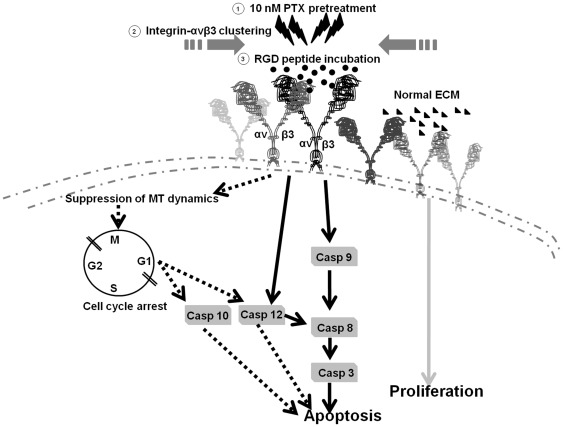
Pathways of caspase cascade and cytochrome c release are involved in U87MG cells treated with RGD peptides in the presence or absence of 10 nM PTX. The low-dose PTX induced the activation of mitotic arrest that is the main cellular mechanism of programmed cell death, and subsequently increased the expression of caspases -10, -12 and -8 for apoptosis (black dashed line). The binding of RGD peptides to integrin-αvβ3 activates caspase -9 then activates downstream caspase -8, -10 and executor caspase -3 (black bold line). Integrin-αvβ3 attached with normal ECM and directed cells to growth or proliferation (the bold gray line). Numbers 1 and 3 represented U87MG cells pre-treated by low-dose PTX or treated by RGD peptides, respectively. Number 2 indicates the recruited phenomenon of integrin-αvβ3 after low-dose PTX pre-treatment. MT: microtubules; ECM: extracellular cell matrix.

In Fig. 6, gene expression of caspase-3 and caspase-8 was increased after the treatment of 10 nM PTX and 40 µM RGD, and caspase-3 was suppressed when it was supplied with caspase-3 or caspase-8 inhibitors. But caspase 8 only was suppressed with caspase-8 inhibitor as well as caspase 9 suppressed with caspase-9 inhibitor neither caspase-3 nor -8 inhibitors. The results of inhibitor neutralized experiment showed that caspase-9 was at the upstream of caspase-8 and -3, and caspase-8 up-regulated caspase-3 undelying the PTX and RGD peptide-induced programmed cell death. The experiments of Fig. 5 and Fig. 6 were not achieved at the same time. Although the values are various among the same treatments, the patterns of fold-change in caspase expression were similar in comparison with control group. Most importantly, this study used 10 nM PTX as a biophysics stressor to modulate integrin-αvβ3 over-expression on the cell membrane of U87MG cells. The up-regulation of integrin-αvβ3 had a potent binding effect on RGD peptides, which competed with matrix molecules and activated programmed cell death during apoptosis. These data offered additional evidence that the up-regulation of integrin-αvβ3 expression increased RGD peptide-induced programmed cell death. In summary, a low dose of 10 nM PTX pre-treatment aroused caspase-10, and the activation of caspase-12 hinged on the up-regulation of integrin-αvβ3 in programmed cell death progress to subsequently stimulate the activation of caspase-9, -8 and caspase-3. Fig. 7 shows the apoptotic pathway of the combination treatment.

Conventional cancer treatments have matured into an effective means for tumor killing in various forms. Even though human glioblastoma U87MG cells are resistant to chemotherapy, there are many methods to destroy human glioblastoma cancer through different cellular mechanisms. The drawbacks of target-specific cancer therapy include the short-lived nature and limited amount of target receptors in tumor cells, which limit therapeutic options and prognoses. Previous studies showed the natural amount of integrin-αvβ3 expressed in various tumor cell membranes, and developed the delivery carriers (liposomes, micelles, nanoparticles) of RGD peptide or its analogue synthesis [44]. The results of this study showed that human glioblastoma U87MG cells expressed integrin-αvβ3, were up-regulated by a pre-treatment of low-dose PTX, and underwent programmed cell death through the activation of caspase-3, -8, -9, -10 and -12. The signal transduction of integrin-αvβ3 correlates to the level of its expression in cells, which can exert through a chemo-drug induction. Further studies should investigate the adjuvant targeting therapeutic strategy in integrin-αvβ3 mediated cellular signaling to determine the naturally occurring integrin-αvβ3 in a variety of cancer cells and ideal stress dosage for its up-regulation.
